# Pseudo (Platelet-type) von Willebrand disease in pregnancy: a case report

**DOI:** 10.1186/1471-2393-13-16

**Published:** 2013-01-17

**Authors:** Neetu Grover, Vincent Boama, Munazzah Rifat Chou

**Affiliations:** 1 Department of Obstetrics & Gynaecology, East Surrey Hospital, Redhill, RH1 5RHT, England, UK; 2 Department of Obstetrics and Gynaecology, Royal Cornwall Hospital, Truro, Cornwall, England, UK

**Keywords:** Von Willebrand, Platelet type, Thrombocytopenia, Pregnancy

## Abstract

**Background:**

Pseudo (platelet-type)-von Willebrand disease is a rare autosomal dominant bleeding disorder caused by an abnormal function of the glycoprotein lb protein; the receptor for von Willebrand factor. This leads to an increased removal of VWF multimers from the circulation as well as platelets and this results in a bleeding diathesis. Worldwide, less than 50 patients are reported with platelet type von Willebrand disease (PT-VWD).

**Case presentation:**

We describe the management of platelet type von Willebrand disease in pregnancy of a 26 year old Caucasian primigravida. The initial diagnosis was made earlier following a significant haemorrhage post tonsillectomy several years prior to pregnancy. The patient was managed under a multidisciplinary team which included obstetricians, haematologists, anaesthetists and neonatologists. Care plans were made for the ante- natal, intra-partum and post-partum periods in partnership with the patient. The patient’s platelet count levels dropped significantly during the antenatal period. This necessitated the active exclusion of other causes of thrombocytopenia in pregnancy. A vaginal delivery was desired and plans were made for induction of labour at 38 weeks of gestation with platelet cover in view of the progressive fall of the platelet count. The patient however went into spontaneous labour on the day of induction. She was transfused two units of platelets before delivery. She had an unassisted vaginal delivery of a healthy baby. The successful antenatal counselling has encouraged the diagnosis of the same condition in her mother and sister. We found this to be a particularly interesting case as well as challenging to manage due to its rarity. Psuedo von Willebrand disease in pregnancy can be confused with a number of other differential diagnoses, such as gestational thrombocutopenia, idiopathatic thrombocytopenia, thrombotic thrombocytopenic purpura and pre-eclampsia; all need consideration during investigations even in a case such as this where the diagnosis of platelet type von Willebrand disease was known before pregnancy.

**Conclusion:**

Management of pseudo von Willebrand disease in pregnancy involves the co-operation of multidisciplinary teams, regular monitoring of platelet levels and factor VIII and replacement as appropriate. This case report highlights this rare condition and the need to exclude all the other differential diagnoses of thrombocytopenia in pregnant women with thrombocytopenia.

## Background

Von Willebrand disease (VWD) is the most common hereditary coagulopathy which results from the deficiency or abnormal function of von Willebrand factor (VWF) which is required for platelet adhesion and aggregation. VWF produced in the endothelium is a multimeric glycoprotein which assists in platelet plug formation by attracting circulating platelets to the site of damage and binds to Factor VIII preventing its clearance from plasma. Presentation varies according to the extent of deficiency and ranges from abnormal menstrual bleeding, bleeding from gums, and nosebleeds to haemathrosis.

The three hereditary types of VWD described are type 1, type 2 and type 3. Type 1 is a quantitative defect and type 2 is a qualitative defect. The severest form is type 3 which is a quantitative defect in which levels of VWF multimers may be undetectable. Type 2B von Willebrand disease was first described in 1980 and is caused by a functionally defective von Willebrand factor (VWF). It is an autosomal dominant bleeding disorder and results from a gain of mutation in the VWF gene, commonly exon 28 coding for the A1 domain.

Closely similar to type 2B is the platelet-type or pseudo- von Willebrand disease (PT-VWD). It is an autosomal dominant disorder caused by gain of function mutations of the VWF receptor on platelets. There is an abnormally enhanced binding of normal VWF to an abnormal platelet glycoprotein Iba (GP1ba) receptor [1].

Mutation in the GP Iba gene (GP1BA) on chromosome 17 results in impairment of the haemostatic function of VWF due to accelerated removal of VWF multimers from the circulation [2].

The Phenotype of PT-VWD is as a result of 4 reported mutations [3-6]:

1) Gly233Val mutation.

2) Met239Val mutation. This plays a critical role in regulation of VWF binding,

3) Gly233Ser mutation,

4) 27 base pair deletion.

Patients with PT-VWD and type 2b VWD have similar phenotypic parameters and clinical symptoms and distinguishing between the two can be a diagnostic challenge. However, these two conditions have different aetiology and require different treatment modalities [7]. A recent case series of platelet type von Willebrand disease in pregnancy highlights the rarity and importance of this condition [8]. The diagnostic criteria for PT-VWD can be challenging and current scientific literatures still debate it. An International registry –based study [9] has reported less than 50 patients worldwide with PT-VWD. This important study also provides the frequency of PT-VWD versus type 2B VWD in addition to the percentage of misdiagnosis of PT-VWD. A clear diagnostic criterion is explained in this publication.

Differentiation of the two conditions has important therapeutic implications. Whereas some patients with type2B VWD can be treated with desmopressin, this is generally avoided as it causes release of abnormal VWF and worsens the bleeding condition. Ideally, patients are treated with platelet transfusion however the majority require treatment with a plasma –derived VWF/factor VIII concentrates and cryoprecipitate. The use of cryoprecipitate in PT- VWD remains contentious, with increasing thrombocytopenia having been reported with these products .Partial correction of plasma VWF level however has been effective in some patients as reviewed by Miller [2]. An individualised approach is suggested for both treatment and prophylaxis in PT-VWD.

## Case presentation

A 26 year old primigravida was referred to the antenatal clinic at 14 weeks gestation with a known diagnosis of pseudo (platelet type) von Willebrand disease. The patient had been asymptomatic with this condition until diagnosis at the age of 16 following excessive haemorrhage immediately after a tonsillectomy. Haematology clinical records indicate the following results at the initial diagnosis: type of GP1BA mutation as Gly233Val, VWF:Ag as 54 IU/dL, VWF:RCO as 38 IU/dL and Factor VIII as 68 IU/dL. The results of VWF levels could not be found and hence are not included. At the time of her antenatal booking, she was asymptomatic and was not undergoing any treatment for her condition. A truly multi-disciplinary team (MDT) was involved in her antenatal, intra-partum and postpartum care which included haematologists, anaesthetists, neonatologists as well as senior obstetricians.

### Antenatal care

The antenatal plan was discussed with the patient during several visits and the MDT. A plan was made for vaginal delivery at term. To facilitate a safe pregnancy and delivery, the following care plan was made:

• Regular antenatal follow up at 24, 28, 32 and 36 weeks gestation

• FVIII and platelet levels to be checked at 24 and 32 weeks gestation

• HLA-matched platelets to be made available on site for delivery

• Any admission associated with the risk of bleeding should prompt a request for the appropriate platelets

#### Intra-partum care plan

• Traumatic delivery should be avoided as the baby has a 50% chance of inheriting the bleeding disorder.

• Regional anaesthesia absolutely contraindicated

#### Post-partum care plan

• Umbilical cord blood for platelet count as well as VWF of the new born

Routine dating scan was performed by a sonographer at 12 weeks gestation. A fetal anomaly scan was performed at 20 weeks gestation. No abnormality of the fetus or pelvic and genital structures was identified. No placental abnormalities were noted. Serial fetal growth scans at 28, 32 and 36 weeks indicated a normal growing fetus.

Throughout the pregnancy the patient became increasingly thrombocytopenic, but remained asymptomatic and clinically well. Other causes of thrombocytopenia in pregnancy such as pre-eclampsia, gestational thrombocytopenia and thrombotic thrombocytopenia were considered and investigated in order to exclude their superimposition on PT-VWD. The following investigations were performed to exclude the above differential diagnosis: a full blood count, clotting screen, a blood film, serum urea, creatinine and electrolytes, 24 hour urine protein collection, liver function tests, regular blood pressure monitoring and a full clinical and neurological examinations. The results of all these investigations remained normal throughout the pregnancy with the exception of the platelet count levels. At 32 weeks the worsening thrombocytopenia (Table 1) prompted a change in the delivery plan. Discussion between the MDT and patient led to the following new plan:

• Induction of labour at 38 weeks in view of worsening thrombocytopenia.

• Cross match 4 units of blood on admission at the time of induction of labour .

• Weekly platelet count until the time of delivery.

**Table 1:** Blood results

**Gestation**	**Haemoglobin g/dl**	**Platelet 10 × 9/l**	**RBC 10 × 12/l**	**HCT**	**MCV**	**MCH**	**Prothrombin time(S)**	**APTT(S)**	**Factor VIII (%)**
Booking	13.6	171	4.55	0.434	88.3	29.9			
24 weeks	12.6	62	3.93	0.35	89.7	32.0			101
28 weeks	13.1	38	4.07	0.37	92.3	32.3			
32 weeks	12.6	17	3.89	0.358	91.2	32.4	13	26	183
34 weeks	12.5	35	3.93	0.365	93.0	31.9	12	25	
36 weeks	13.0	23	4.11	0.380	92.5	31.5			
37 weeks	13.5	40	4.21	0.388	92.3	32.0			
38 weeks	12.9	19	4.05	0.370	92.2	31.9			
Day 1 postpartum	11.1	49	3.47	0.313	90.1	32.0			

The patient was admitted at 38 weeks gestation for induction of labour. She however went into spontaneous labour on the day of her induction. She required 2 units of platelet transfusion before delivery. The active stage of labour lasted about 8 hours. She had an unassisted vaginal delivery of a healthy male baby weighing 3.15 kg with an Apgar score of 9 in 1 minute and 10 in 5 minutes. The total blood loss at delivery was 400ml. The paediatricians attending the delivery performed a comprehensive review of the neonate and also performed the cord blood tests for platelet count and VWF levels. We were informed by the paediatric team that the cord blood tests were all within the normal range.

### Postnatal

The post- partum period was uneventful and she remained clinically well and asymptomatic. Her platelet count was 49 on day 1 postpartum. She was followed up in the community by her general practitioner ( GP) and the haematology team. Her platelet count was 81 and 175 at 2 weeks and 6 weeks postpartum respectively. The patient and her family continue to be under the care of the GP and the haematology team.

## Conclusions

In our patient, there was no diagnostic challenge in distinguishing it from type 2B VWD since the diagnosis of platelet type von Willebrand disease (PT-VWD) had been established pre-pregnancy. Bone marrow biopsy was not performed as we did not find it an appropriate investigation under the circumstances. Von Willebrand factor during the pregnancy were not checked since it was felt by the team that it was not going to affect the clinical management. The challenges faced in this case were determining when to institute platelet transfusion and whether to give cryoprecipitate and also when to induce labour in view of the significant drop in platelet count with advancing gestation. There were also concerns about the wider differential diagnosis of thrombocytopenia including superimposed preeclampsia, gestational thrombocytopenia, idiopathic thrombocytopenia and thrombotic thrombocytopenia which were actively excluded. The rarity of this condition and hence the relative lack of expertise in its management made it more challenging. The involvement of multidisciplinary senior team members resulted in a successful outcome for both the patient and the neonate. The opportunistic counselling that this patient received during her pregnancy also led to the diagnosis under the haematologist of the same condition in both her mother and sibling.

This rare case may have educational value to many obstetricians and haematologists worldwide because it highlights the haemostatic complications with pregnancy. We recommend including PT-VWD in the investigation list for every pregnant woman who presents with thrombocytopenia for the first time. It is the opinion of some haematology experts that Platelet type von Willebrand disease may be found to be more common than current literature indicates if it is sufficiently investigated.

## Consent

Written informed consent was obtained from the patient for publication of this Case report. A copy of the written consent is available for review by the Series Editor of this journal.

## Competing interests

We declare that we have no competing interests as authors of this case report.

## Authors’ contributions

NG was involved in the clinical management of this patient. NG, MRC and VB contributed equally to the write up of this case report. All authors have read and approved the final manuscript.

## Pre-publication history

The pre-publication history for this paper can be accessed here:

http://www.biomedcentral.com/1471-2393/13/16/prepub
